# Editorial: Telomere Dysfunction and Lung Diseases

**DOI:** 10.3389/fmed.2022.861228

**Published:** 2022-03-22

**Authors:** Elizabeth Córdoba-Lanús, Ramcés Falfán-Valencia

**Affiliations:** ^1^Department of Internal Medicine, Dermatology and Psychiatry, Universidad de La Laguna, San Cristóbal de La Laguna, Spain; ^2^Biomarkers Laboratory, Instituto de Enfermedades Tropicales y Salud Pública de Canarias-Universidad de La Laguna, San Cristóbal de La Laguna, Spain; ^3^HLA Laboratory, Instituto Nacional de Enfermedades Respiratorias Ismael Cosío Villegas, Mexico City, Mexico

**Keywords:** telomere, lung, COPD, IPF, ILD, bronchiectasis, TERT, mutations

The concept of aging-associated mechanisms, such as cellular senescence, that contributes to respiratory diseases has emerged in the last decade. Telomeres, which are significant drivers of senescence, are repetitive DNA sequences at the ends of chromosomes critical for maintaining genomic integrity that shorten with each round of DNA replication due to end replication problems (1, 2). Telomere shortening and mutations in genes related to telomere length have been implicated in various lung diseases such as idiopathic pulmonary fibrosis, COPD, emphysema, and lung cancer (3, 4).

This Research Topic presents an update, and the last advances in the basic and translational research on telomere dysfunction concerning the development and progression of certain lung diseases, such as COPD, IPF, and bronchiectasis.

Telomere shortening and mutations in telomere maintenance genes were linked to pulmonary fibrosis. Fibrotic interstitial lung diseases (ILDs) are the first indication for lung transplantation (5); on the other hand, telomere dysfunction has been associated with poor post-transplant outcomes (6). Interestingly, according to the results of Planas-Cerezales et al., post-transplant morbidity is higher in patients with telomere dysfunction and differs according to the elapsed time from transplantation. The study reinforces the benefits of lung transplantation regarding the patient's quality of life if adequate follow-up and management of morbidities are carried out. Similar studies evaluating the relevance of determining telomere length and telomerase gene mutations in the clinical prognosis of subjects with IPF are needed.

Others ILDs include familial pulmonary fibrosis (FPF), a monogenic disease most commonly involving mutations in telomerase reverse transcriptase (TERT) or surfactant protein (SFTP) genes (7, 8). These mutations have been shown to alter lymphocytic inflammatory responses, and FPF biopsies with histological lymphocytic infiltrates have been reported. Since inflammation and fibrogenesis are targeted by different drugs, van Batenburg et al. investigated whether the degree of these two features co-localize or occur independently in different entities of FPF and whether they influence survival compared to sporadic IPF. The results of this investigation show that in sporadic IPF, TERT-related FPF, and SFTP-related FPF, diffuse lymphocyte cell-infiltrates were predominantly present, and lymphocyte aggregates were only present in fibrotic areas. The findings suggest histological similarities between monogenic familial pulmonary fibrosis and sIPF, in accordance with the international guidelines (9). Furthermore, fibroblast foci and percentage of fibrotic lung surface were associated with survival, while no clinically relevant correlation was not observed for lymphocyte aggregates or diffuse lymphocytic infiltration. In addition, the degree of fibrosis, rather than inflammation, correlates with survival. Thus, fibrogenesis is the critical feature for the therapeutic targeting of FPF. Future FPF gene or mutation-specific therapies and treatment with antifibrotic drugs should be tested.

In COPD, a proposed accelerated aging disease, telomere dysfunction, and senescence may act as major players in the impaired cellular regeneration and the pro-inflammatory phenotype (10). In several studies, shortened telomeres were documented in old patients with COPD compared to age-matched controls (11, 12). Interestingly, Casas-Recasens et al. found that young COPD patients also presented short leucocyte telomeres and confirmed that this marker was associated with a worsening of lung function in the old cases but not in the young ones except for those with severe airflow limitation. Unlikely, emphysema was not evaluated in this study. Mitochondrial DNA (mtDNA-CN), the other marker studied, did not behave the same, which sheds some light on one proposed mechanism underlying COPD's pathobiology linked to telomeric shortening. Notably, the study confirms that the relation between TL and COPD is not influenced by cigarette smoke.

Telomere attrition and the genetic alteration in telomere-related genes are well-known causes of pulmonary fibrosis (13, 14). In addition, cellular senescence of alveolar epithelial type 2 cells (AEC2s) has been involved in the genesis of IPF (15). The review by Zhang et al. is focused on the relationship between telomere dysfunction and pulmonary fibrosis mediated by alveolar stem cell AEC2s failure. Authors confront animal models studies and human data to propose a model where senescence of AEC2s leads to the production of a pro-fibrotic niche mediated by SASP (senescence-associated secretory phenotype) conducting to pulmonary fibrosis development. The mechanism suggests an increased mechanical tension and TGF-β signaling loop in the lung, which may lead to the inability to form new alveoli, promoting the development of pulmonary fibrosis due to the significant increase in myofibroblasts. The mini review also contemplates the influence of other factors, such as genetics, epigenetics, occupational exposure, or viral infections, and discusses the importance of differencing genotypes of IPF in the management and progression of patients and for implementing personalized therapies.

Evidence of accelerated aging has been documented in the airways of patients with bronchiectasis (16). The study by Han et al. tried to confirm the existence of accelerated aging in bronchiectasis and its relationship with the severity of the disease by profiling a broad panel of aging markers (telomere length, TERT, SIRT1, Klotho; p16, p21, Ku70, Ku80, TFR2, SOD2) in systemic circulation and different locations of the bronchial epithelium as appropriate. The study proved that bronchiectasis patients present shorter leucocyte telomeres and reduced SIRT1 and Ku80 in peripheral blood compared to controls. Moreover, SIRT1 was found downregulated in peripheral blood and lower airways independent of disease severity and lung function impairment, suggesting an accelerated aging in these patients. Although access to biopsies samples is limited, the lack of age-matched cases and controls should be considered even though not invalidating the presented findings.

Deepening the study of the biology of lung aging will be very useful for the monitoring and treatment of chronic lung diseases such as COPD, emphysema, interstitial lung diseases, or bronchiectasis. Further longitudinal studies are needed to extend beyond our current knowledge of telomere dysfunction and its attrition dynamic to shed more light on the causality of these events with relation to diseases and their clinical impact. Besides, telomere shortening may represent a future therapeutic target. Novel therapeutic strategies to treat and prevent telomere-associated diseases are currently being tested. The applicability of these therapies in a personalized way may help delay the onset or progression of pathological conditions associated with aging.

## Author Contributions

All authors listed have made a substantial, direct, and intellectual contribution to the work and approved it for publication.

## Conflict of Interest

The authors declare that the research was conducted in the absence of any commercial or financial relationships that could be construed as a potential conflict of interest.

## Publisher's Note

All claims expressed in this article are solely those of the authors and do not necessarily represent those of their affiliated organizations, or those of the publisher, the editors and the reviewers. Any product that may be evaluated in this article, or claim that may be made by its manufacturer, is not guaranteed or endorsed by the publisher.
